# Quality of Life in Ugandan Children and Young Adults After Surgery for Congenital Heart Disease: Mixed Methods Approach

**DOI:** 10.5334/gh.1320

**Published:** 2024-04-17

**Authors:** Chloe Searchinger, Hadija Nalubwama, Jafesi Pulle, Rittal Mehta, Hilda Tumwbaze, Rachel Kyarimpa, Rachel Mwima, Emily Atukunda, Bobson Bua, Rachel Sarnacki, Meredith G. Sherman, Michael Oketcho, Meghan Zimmerman, Miriam Nakitto, Chris T. Longenecker, Allison Webel, Amy Scheel, Peter S. Lwabi, Craig A. Sable

**Affiliations:** 1 Princeton University, Princeton, NJ, USA; 2 Division of Cardiology and Global Health Initiative, Children’s National Hospital, Washington, DC, USA; 3 Uganda Heart Institute, Kampala, Uganda; 4 Children’s Hospital at Dartmouth-Hitchcock, NH, USA; 5 Division of Cardiology and Department of Global Health University of Washington School of Medicine, Seattle WA, USA; 6 University of Washington School of Nursing, Seattle WA, USA; 7 Children’s Hospital of Philadelphia, Philadelphia, PA, USA

**Keywords:** Health-related quality of life, Low-income country, congenital heart defect

## Abstract

**Background:**

Health-related quality of life (HRQOL) is a critical issue for patients undergoing surgery for congenital heart disease (CHD) but has never been assessed in a low-income country. We conducted a cross-sectional mixed methods study with age-matched healthy siblings serving as controls at the Uganda Heart Institute.

**Methods:**

One-hundred fifteen CHD pediatric and young adult patients and sibling control participants were recruited. Health-related quality of life was assessed using the Pediatric Quality of Life Inventory Version 4.0 in participants ages 5–17 and 36-Item Short Form Survey for young adults aged 18–25. A subset of 27 participants completed face-to-face interviews to supplement quantitative findings.

**Results:**

Eighty-six pediatric (age 5–17) sibling and parent pairs completed Peds QOL surveys, and 29 young adult (age 18–25) sibling pairs completed SF-36 surveys. One third of patients had surgery in Uganda. Ventricular septal defects and tetralogy of Fallot were the most common diagnoses. Health-related quality of life scores in patients were lower across all domains compared to control participants in children. Reductions in physical and emotional domains of HRQOL were also statistically significant for young adults. Variables associated with lower HRQOL score on multivariate analysis in pediatric patients were younger age in the physical and emotional domains, greater number of surgeries in the physical domain and surgery outside Uganda in the school domain. The only predictor of lower HRQOL score in young adults was surgery outside Uganda in the social domain. Qualitative interviews identified a number of themes that correlated with survey results including abandonment by family, isolation from peers and community, financial hardship and social stigmatization.

**Conclusion:**

Health-related quality of life was lower in Ugandan patients after CHD surgery than siblings. Younger patients and those who had surgery outside of Uganda had lower HRQOL. These data have important implications for patients undergoing CHD surgery in LMIC and have potential to inform interventions.

## Background

Globally, of the 8.5 million infants born each year with a congenital anomaly, 3.1 million of those have congenital heart disease (CHD) [1]. Even though the global birth rate of CHD has remained constant, advances in cardiac surgery have led to a decline in the death rate of those born with CHD by 60.4% in the last two decades [1,2]. As a result, adolescents and adults need life-long care for congenital heart conditions, and health-related quality of life (HRQOL) has emerged as a critically important issue in this population [3,4,5,6,7,8,9].

Despite improvements in cardiac surgery outcomes, there remains a large gap in access to surgical services based on socioeconomic status; many children in low- and middle-income countries (LMICs) continue to experience high morbidity and mortality from CHD [10,11]. Traditionally, low-income countries in sub-Saharan Africa have focused on care of children with unrepaired CHD. Among children, adolescents, and young adults with CHD, reduced HRQOL is well-documented in high income countries (HICs), yet there are very few studies from LMICs [12,13]. A study of cardiac-related HRQOL in Pakistan (a middle-income country) further indicates that HRQOL findings from HICs are not transferable, as they do not take into account resource constraints, or differences in sociodemographic variables, education, and culture [13].

In Uganda, the growth of the Uganda Heart Institute open heart surgery and catheterization programs (established in 2007), in combination with NGO support for sending patients abroad for surgery, has resulted in a substantial number of children (>1000) living after surgical interventional catheterization treatment of CHD in Uganda [10]. This has resulted in a new, expanding and largely understudied group of children and young adults with repaired CHD. As HRQOL has never been assessed in a low-income country, this population provides an opportunity to obtain critical information that will be important for CHD patients in Uganda and help inform care of this population throughout Sub-Saharan Africa.

To investigate HRQOL in Uganda, we conducted a cross-sectional mixed methods quality of life study with age-matched healthy siblings serving as controls to CHD patients at the Uganda Heart Institute in Kampala. After completing HRQOL questionnaires, a subset of CHD patients and their parents were interviewed to qualitatively explore their experience with postoperative CHD. This study’s primary objective was to compare HRQOL in post-surgery patients with CHD to their age-matched healthy siblings. The secondary objective was to determine potential demographic, clinical and environment-related predictors of HRQOL across the age spectrum from childhood through adolescence and into early adult life. We hypothesized that HRQOL would be reduced in participants who have undergone surgery for CHD, and that rigorous assessment of their lived experience could inform the development of HRQOL interventions for this special population.

## Methods

### Recruitment

Using the CHD database at the Uganda Heart Institute and the databases of the Samaritans Purse Children’s Heart Project and Gift of Life International in Uganda, 120 CHD pediatric (age 5–17 years), young adult (age 18–25 years) patients, and 120 sibling control participants (as close in age as possible to repaired CHD patients) were recruited for the quantitative portion of this study. We were able to contact 160 patients/families. These were representative (age, gender, and diagnosis) of a population of approximately 1,000 patients who had undergone surgery for CHD. We chose this paired sibling design to control for potential sociodemographic confounders between children with and without CHD in Uganda. Parents (or guardians) of pediatric patients (*n* = 90) were also recruited to participate.

Participants were between the ages of five and 25 years, had undergone corrective repair of CHD (surgery or catheterization) between the ages of birth and 18 years, or were siblings of those patients currently enrolled in the study. Any patient who had anatomic correction of CHD (as opposed to palliation) was considered to be ‘repaired’ even if there was residual disease (e.g., residual ventricular septal defect, pulmonary stenosis, or pulmonary insufficiency). Healthy siblings were excluded if the age difference was larger than five years or had a chronic medical condition (e.g., HIV and sickle cell disease) that required >3 outpatient visits per year. If there was more than one eligible healthy sibling in a family, the sibling closest in age to the CHD patient was enrolled in the study. Participants were recruited at the Uganda Heart Institute to complete surveys during the months of June and July 2022. Ethics approval was obtained from the Makerere University School of Medicine Research and Ethics Committee (REF: Mak-SOMREC 2021-207) and Ugandan National Council for Science and Technology (REF: HS2115ES). Informed consent was obtained from patients 18 and older and from parents/guardians for patients under 18 by study research staff. Written consents were available in English and Luganda (most common local language).

### Quantitative analysis

For the quantitative assessment of HRQOL, sociodemographic data and clinical information were obtained from a study-specific questionnaire, including age, gender, school-grade, socioeconomic background, household salary, family educational status, occupation, and family structure (Supplemental appendix 1). Participants’ clinical data was retrieved from their medical files, including CHD diagnosis, age at diagnosis and surgery, surgical procedure, residual disease and number of surgical procedures, and CHD Surgical Mortality Risk Score (STAT category 1–5) [14].

Health-related quality of life in participants ages 5–17 was assessed using age-appropriate Pediatric Quality of Life Inventory Version 4.0 Generic Core (PedsQL™ 4.0) questionnaires (examples provided in supplemental appendix 2a and 2b). We chose PedsQL™ 4.0 to be consistent with other HRQOL studies in the congenital heart disease population [5,6,7,12,15]. The PedsQL™ 4.0 tool has been validated with high internal consistency – α = 0.84 (child self-report) and α = 0.87 (parent proxy-report) [16]. Participants/controls and parents/guardians completed the survey separately and results were tabulated separately. Surveys were completed in respondent’s preferred languages, either English or Luganda. It incorporates widely-used questionnaires (appendices 1–2) for children aged 5–17 which assesses HRQOL with four areas of focus: physical functioning (8 items), emotional functioning (5 items), social functioning (5 items), school functioning (5 items) [13]. Each item has five possible responses measuring the extent to which the item was a problem during the past month. The possible responses are measured using a 5-point Likert scale from 0 = never a problem to 4 = almost always a problem. These reverse scaled scores are then transformed to a score of 0–100 to create the overall scaled score and the subcategory scores, with higher scores representing better HRQOL.

To determine the long-term effect of living with surgically corrected CHD, in response to a growing population of surviving CHD patients in which needs such as job access and reproduction are increasingly important, we also assessed HRQOL in young adults (ages 18–25). Because the domains of HRQOL are different for young adults aged 18–25, we used the 36-Item Short Form Survey (SF-36, Supplemental appendix 3), which measures eight domains of HRQOL: physical functioning (10 items); physical role limitations (four items); bodily pain (two items); general health perceptions (five items); energy/vitality (four items); social functioning (two items); emotional role limitations (three items) and mental health (five items) [17]. The questionnaires were conducted in a hospital setting, with participants completing them in a private room provided by the hospital. A study nurse facilitated the process by asking questions and assisting with completion of questionnaires.

Answers to survey data were collected and managed using the REDCap electronic data-capture system hosted at Children’s National Hospital [18]. Baseline data was summarized using mean and standard deviation, and median and interquartile range, where appropriate. Wilcoxon rank sum test was used to compare Peds QOL 4.0 and SF-36 categorical and total scaled scores [12,13,18,19,20]. We used mixed model analyses with random effects to account for family level variation. Multivariable mixed model logistic regression analysis was used to create models for categorical and total scaled HRQOL scores. Variables used in the analyses included age, gender, socioeconomic background, parental education, family structure, cardiac lesion, age at surgery, number of surgeries, STAT scale, location of surgery and cardiac medications as fixed effects, and variation within the family as random effects. Then step wise model selection was performed to understand the important predictors of HRQOL scores among the patients using a p-value of <0.10. Parent scores were excluded from the model due to close overlap with patient self-reported scores. All tests are two-sided and a *p*-value of <0.05 was considered to be statistically significant. All statistical tests were conducted using SAS 9.4 (Statistical Analytical Software).

### Qualitative analysis

Following the quantitative analysis of data, a subset of 27 CHD pediatric and young adult patients participated in face-to-face in-depth interviews to supplement quantitative findings. Three participants with scores in each tertile (low-, middle-, and high-HRQOL) for each age group (ages 5–12, 13–17, 18–25 years) were randomly selected. All participants who were invited for the in-depth interviews agreed to be interviewed. They had all given prior consent during their participation in the quantitative to be contacted for an in-depth interview if selected. Participants under 18 were interviewed together with their parent/guardian, with specific questions asked to parents about their experience [21]. Dyadic interviews for patients under 18 were used to enhance detail of answers; children likely felt more secure with parents by their side and parental input was needed for younger children [21].

Topics addressed during the interview included the patient/parent’s perception of HRQOL related to CHD surgery, identification of the domains of HRQOL that were most important to them during recovery, the areas where the impact of the CHD surgery had been most and least beneficial, the problems/challenges faced, and their perceived needs for the future (Supplemental appendix 4). The interview was guided by themes from the social ecological model (SEM), which has been used in previous studies [12,22,23,24]. The SEM includes variables such as intrapersonal, interpersonal, institutional, community and social-cultural, public policy factors. Males and females were specifically asked about how their heart condition affects plans to marry and have a family. Additional questions about family planning, pregnancy, and childbirth were also included in interviews with females aged 18–25.

Ugandan researchers with public health specialty, and vast experience in qualitative research, conducted individual interviews in Uganda using an in-depth guide (appendix 4) that was reviewed and modified prior to use to assure cultural appropriateness and to promote a conversational tone. The guide later modified to also include unanticipated themes that emerged in the conversation. After conducting the initial six interviews, study team members, inclusive of Ugandan and American researchers, had a discussion of the key themes that were coming out of the interviews. During this discussion, emerging themes of interest from those interviews were adopted in the interview guides to enable more information capturing in the subsequent interviews. Audio recordings of the interviews conducted in the participants’ primary language (Luganda or English) were professionally translated and transcribed into English prior to analysis. Data saturation was reached when it was realized that the same themes were coming out of the interviews repeatedly and thus data collection was stopped. The interview transcripts were reviewed, coded, and analyzed using qualitative descriptive research methods and ATLAS.ti software. [25] Two team members HN & JP, Ugandan public and clinical health specialists respectively with vast experience in conducting and analyzing qualitative research, independently created codes for the first two transcripts and identified recurrent patterns and themes. The team compared and combined their codes and themes into an iterative codebook, which was then used as a guide in coding subsequent transcripts. Frequency of responses and thematically grouped representative quotes are provided in the results sections.

## Results

### Quantitative data

#### Demographics

A total of 115 repaired CHD patients and sibling controls were enrolled (out of 160 patients who were contacted) in the quantitative portion of the study; 86 pediatric (age 5–17) sibling and parent pairs completed Peds QOL surveys, and 29 young adult (age 18–25) sibling pairs completed SF-36 surveys. There were no significant differences in gender mix between patient and control participants in pediatric (57% vs 59% male) or adult (69% vs 55% male) groups. Gender concordance was present in 47% (40/86) of pediatric pairs and 62% (18/29) of young adult pairs. There were also no differences in mean age between patient and control participants in the pediatric (11.6 ± 3.5 vs.11.2 ± 3.1 years) or young adult (20.9 ± 2.2 vs. 21.3 ± 3.7 years) groups. The mean age difference between patient and sibling control participants was 2.6 ± 1.9 years in pediatric and 2.9 ± 1.1 years in young adult groups (p = NS). There was an equal distribution of sibling controls being younger (n = 59) and older (n = 56) than patients. Five pediatric control participants (age range 15–17 years old) completed the SF-36 survey to match the survey completed by their young adult sibling participants (age range 18–20 years old).

Demographic variables for patient participants are shown in Table 1. Family structure and household salary were identical for repaired CHD and control participants. Just under one-third of patients had surgery in Uganda. Ventricular septal defects (VSD) and Tetralogy of Fallot (TOF) were the most common diagnoses which corresponded closely with patients in STAT Categories 1 and 2 respectively. There were no significant differences in case or STAT score distribution between patients who had surgery in Uganda vs. other countries (p = 0.33). The United States and India were the two most common destinations for patients who had surgery abroad.

**Table 1:** Demographics of patient and control participants.


CHARACTERISTICS	N (%)		

**Age**			

5–12 years	46 (40.0%)		

13–17 years	40 (34.8%)		

18–25 years	29 (25.2%)		

	**Total (n = 115)**	**Pediatric (n = 86)**	**Adult (n = 29)**

**Sex**			

Male	69 (60%)	49 (57%)	20 (69%)

Female	46 (40%)	37 (43%)	9 (31%)

**Family structure**			

Single parent family	31 (27%)	22 (26%)	9 (31%)

Two parent family	76 (66%)	64 (74%)	12 (41%)

Others	8 (7%)	0	8 (28%)

**Monthly Household Salary (3600 UGX = $1 USD)**			

0–50,000 UGX	34 (30%)	27 (32%)	7 (24%)

50,000–100,000 UGX	19 (17%)	14 (16%)	5 (17%)

100,000–150,000 UGX	8 (7%)	6 (7%)	2 (7%)

>150,000 UGX	50 (43%)	39 (45%)	11 (38%)

Not reported	4 (3%)	0	4 (14%)

**Location of surgery**			

Uganda	35 (30%)	26 (30%)	9 (31%)

Abroad	79 (69%)	59 (60%)	20 (69%)

Unknown	1 (1%)	1 (1%)	0

**Primary diagnosis**			

Tetralogy of Fallot	37 (32%)	25 (29%)	12 (41%)

Atrial septal defect	14 (12%)	9 (10%)	5 (17%)

Ventricular septal defect	42 (37%)	35 (41%)	7 (24%)

Others	22 (19%)	17 (20%)	5 (17%)

**STAT score(14)**			

1	58 (50%)	45 (52%)	13 (45%)

2	40 (35%)	27 (31%)	13 (45%)

3	6 (5%)	4 (5%)	2 (7%)

4	7 (6%)	6 (7%)	1 (3%)

Not known	4 (4%)	4 (5%)	0

**Number of surgeries**			

1	99 (86%)	75 (87%)	24 (83%)

2	10 (9%)	8 (9%)	2 (7%)

3	3 (3%)	2 (2%)	1 (3%)

4	1 (1%)	0	1 (3%)

Unknown	2 (2%)	1 (1%)	1 (3%)


Abbreviations: STAT score; The Society of Thoracic Surgeons – European Association for Cardio-Thoracic Surgery Congenital Heart Surgery Mortality Categories; UGX: Ugandan Schillings; USD: United States Dollar.

#### QOL survey performance

Quality of life scores in patients were lower across all domains compared to control participants in children taking the Peds QOL Survey (Table 2). Differences were statistically significant across all pediatric age groups in physical, emotional, and social domains, in physical and school domains in children age 5–12 and in social domains in children age 13–17. Peds QOL Surveys completed by parents showed nearly identical results with observed differences in same domains as those in self-reported answers. Reductions in physical and emotional domains of HRQOL were also statistically significant for young adults living with corrected CHD compared with their sibling pairs (Table 3).

**Table 2:** HRQOL scores in children – patients vs sibling controls (self report).


DOMAIN	PATIENTS	SIBLING CONTROL PARTICIPANTS	P-VALUE

**All Children (5–17 years)**			

Physical score	88.74 ± 15.79	95.13 ± 8.77	**0.0003**

Emotional score	89.77 ± 14.43	92.79 ± 10.42	**0.0444**

Social score	90.35 ± 16.87	94.65 ± 10.59	**0.0178**

School score	82.57 ± 17.54	86.08 ± 14.08	0.2210

**Age (5–12 years)**			

Physical score	89.95 ± 18.02	96.81 ± 6.59	**0.0097**

Emotional score	92.50 ± 16.01	94.52 ± 10.49	0.3605

Social score	90.98 ± 18.22	94.52 ± 9.81	0.3962

School score	80.22 ± 18.98	87.45 ± 13.33	**0.0423**

**Age (13–17 years)**			

Physical score	87.34 ± 12.85	92.56 ± 10.93	0.0815

Emotional score	86.63 ± 11.79	90.15 ± 9.88	0.1818

Social score	89.63 ± 15.38	94.85 ± 11.84	0.0585

School score	85.35 ± 15.46	83.97 ± 15.11	0.6467


Abbreviation: HRQOL: Health Related Quality of Life.

**Table 3:** HRQOL scores in young adults – patients vs sibling controls (self report).


DOMAIN	PATIENT	SIBLING	P-VALUE

**Adults – SF 36**			

Physical score	87.48 ± 11.18	92.07 ±10.48	**0.0394**

Emotional score	71.31 ± 16.32	79.03 ± 14.84	**0.0401**

Social score	81.07 ± 21.02	86.24 ± 20.94	0.1291

General score	75.69 ± 19.90	80.00 ± 16.96	0.2470

Pain	73.41 ± 25.98	82.00 ± 24.68	0.2119

Energy fatigue	68.28 ± 15.66	73.10± 17.13	0.1711

Physical (Role limitation)	81.90 ± 31.27	93.97 ± 17.24	0.0519

Emotional (Role limitation)	89.62 ± 26.95	88.52 ± 25.65	0.2943


Abbreviation: SF: Short Form Survey.

#### Predictors of HRQOL

Variables associated with lower HRQOL score on multivariate analysis in pediatric patients were younger age (at time of study) in the physical and emotional domains, greater number of surgeries in the physical domain and surgery outside Uganda in the school domain. The only predictor of lower HRQOL score on multivariate analysis in young adult patients was surgery outside Uganda in the social domain (Table 4). On direct comparison (Supplemental Table), patients who had surgery in Uganda (*n* = 35) had higher scores in several domains when compared to patients who had surgery abroad (*n* = 79, primarily in India and United States. There were no significant differences between patients who had one surgery (n = 99) and those that had two or more (n = 14) surgeries.

**Table 4:** Multivariate predictors of hrqol in patients (based on self report patient scores).


AGE GROUP	TOOL	DOMAIN	VARIABLE	OR (95% CI)	*P-VALUE*

Children	Peds QOL	Physical	Age group (1 vs 2)	2.749 (1.041–7.260)	*0.0413*

			Number of surgeries	5.241 (1.14–24.07)	*0.0332*

Children	Peds QOL	Emotional	Age group (1 vs 2)	4.263 (1.91–9.51)	*<0.0001*

Children	Peds QOL	School	Surgery location (Abroad vs Uganda)	3.562 (1.292–9.826)	*0.014*

Young adult	SF-36	Social	Surgery location (Abroad vs Uganda)	8.385 (1.298–54.181)	*0.0255*


Abbreviations: HRQOL: Health Related Quality of Life; QOL: Quality of Life; SF: Short Form Survey.

### Qualitative data

Qualitative interviews were performed with 27 patients. There were nine participants in each of the three group and 11/27 were females (3, 4, and 4 females in the 5–12, 13–17, and 18–25 age groups respectively). The most common themes and frequency of mentioning those themes and representative quotes are shown in Table 5.

**Table 5:** Thematic responses by patient participants and parents from qualitative interviews t.


SCHOOL PERFORMANCE IMPACT	FREQUENCY	TERTILE			REPRESENTATIVE QUOTES

		H	M	L	

**Before CHD surgery**					‘*He doesn’t miss school since they inserted the metal inside him to open the blocked vessels. Before that, he would vomit that you couldn’t take him to school in that condition.’ – **Parent to Participant 38P (8 yrs, MQOL)*** ‘*Right now I spend more time at school. I no longer fall sick as often like in the past ever since I had my second operation. Now I only return home if my mother calls at school asking for me to go and visit the hospital.’ –* ***Participant 37P (15 yrs, LQOL)***

Affected	21	6	8	7	

Unaffected	3	2	1	0	

**After CHD surgery**					

Improved performance and attendance	14	8	4	2	

**PHYSICAL FUNCTIONALITY IMPACT**					

**Before CHD surgery**					‘*I never used to play. I used to be in one place watching others play. I also used to arrive late at school. I didn’t have the energy to walk.’ –* ***Participant 23p (24 yrs, HQOL)*** ‘*He (patient) is fine because he can go and fetch some water, he can graze the goats, milk the cow and he can do some work at home. He can ride a bicycle, you can send him to go to the shop and bring something and in a short time he is back but he could not before (CHD surgery). I feel very happy.’ –* ***Parent to Participant 25p (16 yrs, HQOL)*** ‘*There is nothing that my health has stopped me from doing because recently, I took a choice to start working out and it hasn’t affected my health in any way. I feel okay; I can do anything as long as I choose to do it.’ –* ***Participant 42 (24 yrs, HQOL)***

Inability to do daily chores	7	4	1	2	

Inability to participate in sports/games	10	4	3	3	

**After CHD surgery**					

Improved ability to do daily chores	19	7	8	4	

Improved ability to participate in sports/games	12	5	5	2	

**EMPLOYMENT IMPACT**					

Worried/unable to get desired jobs	5	3	2	0	‘*I at one point, went to a delivery company after my form 4 and they asked me about my condition, I told them that I had a heart surgery when I was young. They told me that I couldn’t get a job there because they lift heavy things. So, that affects finding employment. I am finishing my mechanics course this year and I will start searching for a job however, I know that I will be affected and I will end up getting a job from someone that I know*.’ ***– Participant 79p (21 yrs MQOL)*** ‘*I don’t think it will affect me because I am really fine. It would affect me if I am getting any challenges with my health right now, that’s how it would be tough but I don’t think that it will affect me.’ –* ***Participant 42 (24 yrs HQOL)***

Not worried/able to get desired jobs	5	1	2	2	

**SOCIAL LIFE IMPACT**					

**Partner relationships impact**					

Affected/Worried about marital relationships	2	0	0	2	‘*I might get married to a stubborn woman who might start disturbing me and then stress me. So, that makes me feel like, even if I do not get married soon, let me first live a single life such that my life and health stabilize.’ –* ***Participant 90P (19yrs, LQOL)*** ‘*They (students) would say that I am disabled and I didn’t sexually grow correctly. Yeah, they used to say that I can’t give birth’ –* ***Participant 30p (22rys, LQOL)*** ‘*For them, (community members) they think that I cannot get married. They say things like, “now who can marry someone who had a heart disease?” Okay, they call me lame.’ –* ***Participant 13p (20yrs, LQOL)*** ‘*I don’t think it affects my marriage plans or my relationship because right now, I’ve been in a relationship for 5 years and it hasn’t affected my relationship. So, I don’t think it will affect marriage’ –* ***Participant 42 (24 yrs, HQOL)***

Not affected/worried about marital relationships	8	3	2	3	

**Childbearing impact**					

Affected/worried about childbearing	3	2	0	1	‘*That thing is on my mind and I do not know why it disturbs me – that I will never give birth! I just feel bad about it.’ –* ***Participant 13p (20yrs, LQOL)***

Not affected/worried about childbearing	7	3	1	3	

**Community relationship impact**					

**Before CHD surgery**					‘*You know at school I was isolated at first… students would be afraid of me because when they took me to that school, children were told, “This girl had a heart problem. So, if you push her and she falls, I don’t know whether your parents will get money to put her in the airplane and fly her out!” So, nobody would play with me.’ –* ***Participant 13P (20yrs, LQOL)*** ‘*Every time a teacher was going to cane me, they (friends) would quickly tell him/her not to. They would say, “Let him be. His health is confused.”’ –* ***Participant 79p (21 yrs, MQOL)*** ‘*The people in the community didn’t mistreat me but the children would mock me before the operation. Some would tell me that I have a bicycle heart. But since I was operated on, I don’t hear those things anymore… they would say that I am weak and don’t have power. “She’s sickly and her heart isn’t real but a bicycle heart.”’ –* ***Participant 66p (aged 18 yrs, HQOL)***

Faced stigmatization/rejection	9	3	3	3	

**After CHD surgery**					

No longer faced stigmatization/rejection	2	1	1	0	

**HEALTH IMPACT**					

**Emotional health impact**					

**Before CHD surgery**					‘*I used to be alone and envy or be jealous of other people doing things that I couldn’t do, basically, I won’t say that there were people isolating me because I was self-isolating myself. I was mentally perturbed At school, I was kind of segregated.’ –* ***Participant 30p (22 yrs, MQOL)***

Short tempered	3	0	0	3	

Self-isolation/consciousness	4	2	1	1	

**After CHD surgery**					

No longer self-isolates	1	0	1	0	‘*After the operation, I regained my humility. I was like, “I am now feeling better! You never know; I might be a professor of tomorrow or a doctor of tomorrow!” So, my heart relaxed again. Even my friends started coming back in my life because I had avoided them since they looked happy and yet I was sad. I remember they would play football and yet I was just sitting there watching! They would run around but I was just sitting!’ –* ***Participant 90p (19 yrs, MQOL)***

**Psychological health Impact**					

**Before CHD surgery**					‘*I randomly just get depressed on my own. When I fall sick, like that time when I used to breathe and feel pain in the chest, my mind just told me, “you are about to die – you are about to die” and then, “you won’t give birth!”’ –* ***Participant 13P (20 yrs, LQOL)*** ‘*I used to get mental problems, stress and self-isolation. I would spend the entire day indoors and not wanting to talk to anyone (before surgery) … I am (now) emotionally doing well and mentally am doing fine that’s what I can say. It is not as bad as before I went for the surgery.’ –* ***Participant 30p (22 yrs, MQOL)***

Worry/panic/despair/frustration	13	4	4	5	

Forgetfulness	2	0	1	1	

Depression	4	2	0	2	

**After CHD surgery**					

No more worries/panics/frustration	15	4	7	4	

**Physical growth and development**					

**Before CHD surgery**					‘*Before the surgery, he was stunted and dark skinned but the Indians called it being “purple.” He was like a child who got burnt and wasn’t growing any hair. He wasn’t growing in any way, he had swollen fingers and feet and he also couldn’t move from here to there. Currently, since the operation you can see that he growing and he looks good. However, he has to get another operation.’ –* ***Parent to Participant 6P (8 yrs, LQOL)*** ‘*She recovered very well. She has never gotten any problem, not even falling sick. Otherwise, in the past, she used to fall sick so often that we were always admitted. But ever since her surgery, we have never been admitted again. Except the normal illnesses like malaria.’ –* ***Parent to Participant 75 (17 yrs, MQOL)***

Failure to thrive	7	2	2	3	

Sickly	11	6	2	3	

**After CHD surgery**					

Reduced or no ill episodes experienced	18	7	7	4	

Enjoys normal growth/thriving	8	4	2	2	


Abbreviations: HQOL: High Quality Life Tertile; LQOL: Low Quality of Life Tertile; MQOL: Middle Quality of Life Tertile; yrs: years.

A summary of qualitative findings by theme is provided here:

#### Theme 1. School performance impact

Patient education was reported to be affected profoundly by CHD. Congenital heart disease consumed financial resources, especially those led by women who were abandoned by their husbands due to CHD. Reports of delayed start of school and missing school days due to sickness were made by both the patient and their parents/guardians. This improved when the patients received surgery. Despite the improved school attendance, learning difficulties persisted after surgery.

#### Theme 2. Physical functioning impact

**Ability to do daily chores and participate in sports/games:** Congenital heart disease profoundly impacted the physical functioning of patients especially before surgery. It limited patients’ mobility and meaningful participation in daily household chores as well as sports and games. The inability to engage actively in sports or play with other children was reported by patients below 15 years as greatly affecting their happiness. Sports participation after surgery contributed to happiness, completeness, and normalcy. For older patients, regaining the ability to engage in daily work after surgery allowed for a return to normal living. Patients who required additional surgery reported still having functional challenges including tiredness and inability to participate in sports.

#### Theme 3. Employment impact

Many CHD patients interviewed talked about their limitations in the kind of jobs they could engage in due to their compromised health state. Parents/guardians attempted to direct patients towards non-strenuous occupations. Many young adult patients mentioned that they would avoid jobs that require using a lot of energy. During job interviews, some avoided disclosing their CHD history for fear of not being hired. Some CHD patients reported no limitations after surgery and to be able to perform any job.

#### Theme 4. Social life impact

**Partner relationships and childbearing impact:** Many young adults felt that CHD did not affect their current or future relationships with their partners or marriages; they were getting emotional support from their partners after disclosing their CHD history. However, some young adults and older children, primarily females, reported concern about not having families and bearing children who could end up with the same condition. They reported stigmatizing statements from family and community members regarding their limited chances of getting married due to CHD. Male CHD patients were more confident about their current and future relationships and marriages. A few male respondents preferred to delay engaging in relationships due to fear of stress compromising their health and some experienced demeaning statements from colleagues about their sexual functionality.

**Community relationship impact:** Congenital heart disease patients reported rejection, stigmatization, and discrimination due to undesirable physical appearance before surgery. After surgery, many reported acceptance by families and communities while a minority still reported being stigmatized by community members. Some CHD patients continued to be labeled as lame or disabled and/or still received much-undesired attention from family members, schoolmates, friends, and other community members.

#### Theme 5: Health impact

**Emotional health impact:** Congenital heart disease impacted the patient’s emotional stability often making them have short tempers, irritability, depression, self-isolation, and self-consciousness. Many CHD patients and parents or guardians reported that before surgery, CHD patients did not have the energy to fight or defend themselves against people who provoked them, which made them irritable and short-tempered. Others preferred to stay in isolation to avoid stress and feelings of not belonging to the groups around them. Improvement in emotional stability was reported by CHD patients after surgery with many reporting improved self-confidence not needing to isolate. However, the surgery scar continued to make them self-conscious and selective in what they wear and who they socialize with.

**Psychological health Impact:** Many patients reported constant fear, uncertainty, panic, despair, and stress before surgery. Worries about being rejected by schoolmates and the possibility of not completing their education or dying, given the severity of CHD, were often mentioned. These psychological symptoms were reported to have resolved by most of the patients after surgery, but less so by those who still had symptoms or were in need of additional surgeries.

**Physical growth and development impact:** Study respondents, especially parents or guardians, expressed their joy due to the transformation they witnessed in physical growth and appearance resulting from the CHD surgery.

**Ill health episodes impact:** Most patients and parents or guardians reported a dramatic change in the health of the CHD patients after surgery, either no illness or minor illnesses that could be easily managed with medication.

## Discussion

This is the first study to assess the impact of CHD surgery on HRQOL in patients and families in a LMIC. We found that HRQOL was lower in all four domains in children and in two of eight domains in young adults. The most striking differences across all age groups were in the physical domain. Younger age and having surgery outside of Uganda were the most consistent factors associated with lower HRQOL among CHD patients, independent of socioeconomic status, diagnosis, and STAT risk category. Qualitative interviews provided important additional perspectives with many of the patients reporting significant limitations and stigmatization prior to surgery and improvement after surgery. Specific concerns around pregnancy and parenting were expressed by adolescent and young adult females. Parents also were profoundly impacted by their child’s CHD in several domains, including family and community relationships, and financial hardships.

Although surgery has improved survival for CHD, it is generally reparative rather than curative, with common postoperative issues including respiratory infections, frequent hospitalizations, poor exercise tolerance, stroke, arrhythmias, heart failure, compromised cardiac function, higher risk pregnancy and child birth, employment challenges and need for further medical intervention [4,5,8,15,26,27]. Health-related quality of life assessment following cardiac treatments is further recommended by the Centers for Disease Control and Prevention and the American Heart Association [28]. PedsQL™ 4.0 has well-established reliability in past HRQOL and CHD-specific studies [13]. The tool has been validated in more than 90 country/language combinations [13,20,29]. The impact of CHD surgery on HRQOL is necessary to consider, as clinical measures may not fully encompass disease symptoms, treatment burden, functional limitation and adaptation to life experience [5,15,26].

Traditionally, countries in sub-Saharan Africa have focused on care of children with unrepaired CHD and not on HRQOL in children, adolescents, and young adults with repaired CHD [12,13]. Children in LMIC continue to experience high morbidity and mortality from CHD [10,11]. In low socio-demographic index (SDI) regions, CHD mortality rates have declined by only 42.1% compared with a 71.3% decline in high-SDI countries [1]. Over 90% of the world’s children do not have access to cardiac surgery [30]. In Sub-Saharan Africa’s population of approximately one billion, there is an estimated need for 350,000 open heart surgeries (OHS) per year [30]; one-third of which are for CHD. Based on available cardiac surgery services, only 3% of children with CHD in sub-Saharan Africa who need cardiac surgery actually receive it [30]. Much can be learned from Uganda as microcosm for sub-Saharan Africa. With the third highest birthrate in the world, estimates range from 8,300 to 36,000 babies born with CHDs in Uganda annually, of which 25% are serious enough to require cardiac interventions [10]. Our data underscore the need for increased investment in tertiary interventional capacity for patients with CHD. While beyond the scope of this paper; further analysis of the burden of CHD from an economic standpoint is crucial to inform public health policy in Uganda and other LMICs.

The overlap of poverty, high fertility rates, limited access to care, and more traditional gender roles results in a unique profile of CHD in LMIC. Therefore, cardiac-related HRQOL specifically in LMIC must be further investigated [30]. This population is at risk for negative impacts on HRQOL due to their unmet need for lifelong care for residual CHD symptoms in resource-constrained environments with decreased access to healthcare services.

The study of HRQOL in Uganda is an opportunity to address a large gap in our understanding of long-term patient outcomes of CHD patients after heart surgery in LMICs, particularly as children mature into adults and girls become of childbearing age. Worse HRQOL outcomes (such as cognitive function deficits and diminished exercise tolerance) have been reported in surgically corrected CHD children and adolescents in HIC as compared to their peers without CHD, [4,5,6,8,9,20,31] but there is scant research from LMIC. Therefore, it is imperative to elucidate the complexity of HRQOL in LMIC and identify possible interventions to improve the lives of children living with CHD as they transition to adulthood [10].

As with other studies, HRQOL was lower among participants with repaired CHD compared to control participants in Uganda. Absolute HRQOL scores in our cohort of CHD patients were slightly higher than in studies from middle- and high-income countries, while scores in control participants were similar [7,13,32]. We attribute this to our Ugandan cohort having less severe CHD than those in other studies. A number of relevant cultural and sociodemographic HRQOL predictors were identified in a previous cardiac-HRQOL study conducted in Pakistan, such as gender and cultural attitudes and expectations of women [13,26]. In the Pakistan study, HRQOL was worse for patients who were on cardiac medications, had complex CHD, longer cardiopulmonary bypass time, re-operations and female sex. Having more operations was the only one of these factors associated with HRQOL in our study. This may be in part related to differences in disease complexity between patients undergoing surgery in Uganda and Pakistan. HRQOL scores were lowest in emotional functioning domains in both Pakistan and Uganda studies. Our qualitative interviews identified several important themes in this area including abandonment by family, isolation from peers and community, and social stigmatization.

Patients who had surgery in Uganda reported higher HRQOL than those who had surgery abroad (primarily in the United States and India) across several domains. This was not explained by differences in complexity of diagnosis, surgical risk category, or socioeconomic status. Qualitative interviews included comments about the uncertainty of traveling abroad, loss of employment, financial hardship, and perception about lack of available medications and care needed upon returning to Uganda. Although not explicitly mentioned by participants in interviews, it is possible that there is more social stigmatization and community isolation when the patient and parent are away for an extended period of time. These patients may have the perception that they were treated abroad because their condition could not be handled in Uganda and that upon return, they might not be afforded the same quality of care. They may prefer to be followed-up by the team that operated on them. More detailed preparation for patients and caregivers before and after the surgery on how follow-up care will be handled could help to address this issue. Peer groups of these patients for sharing their experiences and challenges is also something to consider [18].

Both our study and the one from Pakistan highlighted concerns of young women around childbirth and motherhood. A previous qualitative Ugandan study that surveyed women of childrearing age with corrected rheumatic heart disease (RHD) cited pre-existing heart disease in pregnant women as one of the greatest risk factors for obstetric mortality [27]. The women participants of that study recounted issues such as a lack of knowledge about pregnancy risk and negative stigma experienced for being deemed unfit to reproduce, suggesting that ‘health programs targeting health disease in LMICs must pay special attention to the needs of women of child-bearing age’ [27]. To target areas of potential intervention, more focused assessment of HRQOL in women of childbearing age with CHD in low-income countries is an important next step.

Our study has several important limitations. We chose sibling control participants who were not the exact same age as repaired CHD participants and half were the opposite gender. The gender mismatch likely limited our quantitative assessment of the impact of reproductive issues in young adult participants. Gender inequality could have been an additional source of differences in HRQOL between patient and control participants. Additionally, control participants may have been impacted by stress of having a family member with CHD. However, this methodology likely introduced fewer confounding variables than if we had selected non-sibling controls. In fact, using siblings might underestimate the impact of CHD on HRQOL. Our population did not include many patients with multiple surgeries, complex CHD, or higher STAT category operations, which may limit comparability to other published studies. However, our population is likely representative of those patients undergoing CHD surgery in low-income countries (mostly ventricular septal defects and tetralogy of Fallot). Our study was not powered to comprehensively study the effects of HRQOL on childbearing in young women. Additionally, our limited sample size prevented us from doing multiple testing corrections which would have further reduced statistical power. This could be a source of Type I error. Our separate analysis of parent reported survey scores did not add additional information other than to validate self-reported scores.

## Conclusion

This is the first study to evaluate HRQOL in children and young adults living with repaired CHD in a low-income country. Health-related quality of life was lower in both children and young adults. Younger patients and those who had surgery outside of Uganda had lower HRQOL across several domains. Qualitative interviews highlighted several important themes, most notably significant physical and social limitations felt by children prior to surgery and stigmatization, community isolation, and financial hardship experienced by parents of patients. Adolescent and young adult women expressed concerns about pregnancy and motherhood. These data have important implications for patients after undergoing CHD surgery in LMIC and have the potential to inform interventions. While many of these issues are present in middle- and high-income countries, poverty, cultural attitudes, and expectations of women may magnify these issues and mandate development of unique interventions.

## Data Accessibility Statement

Raw quantitative data from HRQOL surveys and qualitative data from transcribed transcripts (Atlas.ti) is available upon reasonable request.

## Additional Files

The additional files for this article can be found as follows:

10.5334/gh.1320.s1 Supplementary Table. HRQOL Scores by Location of Surgery.

10.5334/gh.1320.s2 Supplemental Appendices. Appendix 1 to 4.
