# Supplementary material for: Quality of Life in Ugandan Children and Young Adults After Surgery for Congenital Heart Disease: Mixed Methods Approach
Source: Glob Heart. 2024 Apr 17;19(1):36. doi: 10.5334/gh.1320 (PMC11025577; doi:10.5334/gh.1320)
Supplement: Supplementary Table. — HRQOL Scores by Location of Surgery. [file gh-19-1-1320-s1.pdf]

Supplemental Table: HRQOL Scores by Location of Surgery

**Peds QL 4.0 (Pediatric Patients)**	**Uganda**	**Abroad**	***p-value***
Physical score	89.66 ± 11.82	88.14 ± 17.39	*0.4892*
Emotional score	87.12 ± 11.50	90.93 ± 15.60	*0.184*
Social score	90.77 ± 16.41	90.00 ± 17.30	*0.0985*
School score	89.80 ± 11.94	79.27 ± 18.81	***0.0117***
**SF-36 (Young Adults Patients)**			
Physical score	92.00 ±9.07	85.45 ± 11.65	*0.078*
Emotional score	78.22 ± 14.85	68.20 ± 16.34	*0.0592*
Social score	94.44 ± 11.02	75.05 ± 21.84	***0.0119***
General score	86.67 ± 11.99	70.75 ± 20.98	***0.0311***
Pain	88.67 ± 17.35	66.55 ± 26.61	***0.0168***
Energy Fatigue	71.67 ±18.71	66.75 ±14.35	*0.319*
Physical (Role limitation)	97.22 ± 8.33	75.00 ± 35.36	*0.0558*
Emotional (Role limitation)	100.00 ± 0.00	84.95 ± 31.57	*0.0896*
