# Supplementary material for: Quality of Life in Ugandan Children and Young Adults After Surgery for Congenital Heart Disease: Mixed Methods Approach
Source: Glob Heart. 2024 Apr 17;19(1):36. doi: 10.5334/gh.1320 (PMC11025577; doi:10.5334/gh.1320)
Supplement: Supplemental Appendices. — Appendix 1 to 4. [file gh-19-1-1320-s2.pdf]

Appendix 1: Background Questionnaire

Intake Form

Record ID: ______________________________

Consent signed:

- ☐ Yes
- ☐ No

Status:

- ☐ Patient
- ☐ Control

Demographics (to be filled out by participants)

Date: ______________________________

Surname: ______________________________

First name: ______________________________

Age group:

- ☐ Children (5-12)
- ☐ Adolescent (13-17)
- ☐ Young Adult (18-25)

Age: ______________________________

School grade (of child):

Sex:

- ☐ Male
- ☐ Female

Village: ______________________________

Parish: ______________________________

District: ______________________________

Subcounty: ______________________________

Telephone number/whose phone number: ______________________________

Family Structure: ______________________________

- ☐ One Parent
- ☐ Two Parent
- ☐ Other

Highest Family Parent Education:

- ☐ No degree

- ☐ High school degree
- ☐ College degree
- ☐ Graduate school degree

Parent job/source of income: ____________________________________

Salary (monthly):

- ☐ 0 – 50,000 UGX
- ☐ 50 – 100,000 UGX
- ☐ 100,000 – 150,000 UGX
- ☐ Greater than 150,000 UGX

<u>CHD Background (to be filled out by staff)</u>

CHD diagnosis: ____

Age at surgery: ____

Number of surgical procedures: ____

Location of surgery:

- ☐ Uganda
- ☐ Abroad (if so, where _________________________)

STAT score: ____

Current cardiac medication: ____

Current cardiac symptoms: ____

Appendix 2a: PedsQL Version 4.0 Child Report

Quality Of Life

Record ID ______________________

**Health and Activities (in the past one month)**	
It has been hard to walk for more than five minutes:	◯ Never<br>◯ Almost Never<br>◯ Sometimes<br>◯ Often<br>◯ Almost Always
It has been hard to run:	◯ Never<br>◯ Almost Never<br>◯ Sometimes<br>◯ Often<br>◯ Almost Always
It has been hard to do sports activity or exercise:	◯ Never<br>◯ Almost Never<br>◯ Sometimes<br>◯ Often<br>◯ Almost Always
It has been hard to lift something heavy:	◯ Never<br>◯ Almost Never<br>◯ Sometimes<br>◯ Often<br>◯ Almost Always
It has been hard to bathe or wash by myself:	◯ Never<br>◯ Almost Never<br>◯ Sometimes<br>◯ Often<br>◯ Almost Always
It has been hard to help out around the house:	◯ Never<br>◯ Almost Never<br>◯ Sometimes<br>◯ Often<br>◯ Almost Always
I hurt or ache:	◯ Never<br>◯ Almost Never<br>◯ Sometimes<br>◯ Often<br>◯ Almost Always
I have low energy:	◯ Never<br>◯ Almost Never<br>◯ Sometimes<br>◯ Often<br>◯ Almost Always

Feelings (in the past one month)	
I feel afraid or scared:	◯ Never<br>◯ Almost Never<br>◯ Sometimes<br>◯ Often<br>◯ Almost Always
I feel sad:	◯ Never<br>◯ Almost Never<br>◯ Sometimes<br>◯ Often<br>◯ Almost Always
I feel angry:	◯ Never<br>◯ Almost Never<br>◯ Sometimes<br>◯ Often<br>◯ Almost Always
I have trouble sleeping:	◯ Never<br>◯ Almost Never<br>◯ Sometimes<br>◯ Often<br>◯ Almost Always
I worry about what will happen to me:	◯ Never<br>◯ Almost Never<br>◯ Sometimes<br>◯ Often<br>◯ Almost Always

Getting Along with Others (in the past one month)	
I have trouble getting along with other kids:	◯ Never<br>◯ Almost Never<br>◯ Sometimes<br>◯ Often<br>◯ Almost Always
Other kids do not want to be my friend:	◯ Never<br>◯ Almost Never<br>◯ Sometimes<br>◯ Often<br>◯ Almost Always
Other kids tease me:	◯ Never<br>◯ Almost Never<br>◯ Sometimes<br>◯ Often<br>◯ Almost Always
I cannot do things that other kids my age can do:	◯ Never<br>◯ Almost Never<br>◯ Sometimes<br>◯ Often<br>◯ Almost Always

It is hard to keep up when I play with other kids:	◯ Never<br>◯ Almost Never<br>◯ Sometimes<br>◯ Often<br>◯ Almost Always

School (in the past one month)

It is hard to pay attention in class:	◯ Never<br>◯ Almost Never<br>◯ Sometimes<br>◯ Often<br>◯ Almost Always
I forget things:	◯ Never<br>◯ Almost Never<br>◯ Sometimes<br>◯ Often<br>◯ Almost Always
I have trouble keeping up with my schoolwork:	◯ Never<br>◯ Almost Never<br>◯ Sometimes<br>◯ Often<br>◯ Almost Always
I miss school because of not feeling well:	◯ Never<br>◯ Almost Never<br>◯ Sometimes<br>◯ Often<br>◯ Almost Always
I miss school to go to the doctor or hospital:	◯ 1. Never<br>◯ 2. Almost Never<br>◯ 3. Sometimes<br>◯ 4. Often<br>◯ 5. Almost Always

Appendix 2b: PedsQL Version 4.0 Parent Report

Quality Of Life

Record ID ______________________________

Health and Activities (in the past one month)

It has been hard for my child to walk for more than five minutes:

- ◯ Never
- ◯ Almost Never
- ◯ Sometimes
- ◯ Often
- ◯ Almost Always

It has been hard for my child to run:

- ◯ Never
- ◯ Almost Never
- ◯ Sometimes
- ◯ Often
- ◯ Almost Always

It has been hard for my child to do sports activity or exercise:

- ◯ Never
- ◯ Almost Never
- ◯ Sometimes
- ◯ Often
- ◯ Almost Always

It has been hard for my child to lift something heavy:

- ◯ Never
- ◯ Almost Never
- ◯ Sometimes
- ◯ Often
- ◯ Almost Always

It has been hard for my child to bathe or wash by themself:

- ◯ Never
- ◯ Almost Never
- ◯ Sometimes
- ◯ Often
- ◯ Almost Always

It has been hard for my child to help out around the house:

- ◯ Never
- ◯ Almost Never
- ◯ Sometimes
- ◯ Often
- ◯ Almost Always

My child hurts or aches:

- ◯ Never
- ◯ Almost Never
- ◯ Sometimes
- ◯ Often
- ◯ Almost Always

My child has low energy:

- ◯ Never
- ◯ Almost Never
- ◯ Sometimes
- ◯ Often
- ◯ Almost Always

**Feelings (in the past one month)**	
My child feels afraid or scared:	◯ Never<br>◯ Almost Never<br>◯ Sometimes<br>◯ Often<br>◯ Almost Always
My child feels sad:	◯ Never<br>◯ Almost Never<br>◯ Sometimes<br>◯ Often<br>◯ Almost Always
My child feels angry:	◯ Never<br>◯ Almost Never<br>◯ Sometimes<br>◯ Often<br>◯ Almost Always
My child has trouble sleeping:	◯ Never<br>◯ Almost Never<br>◯ Sometimes<br>◯ Often<br>◯ Almost Always
My child worries about what will happen to them:	◯ Never<br>◯ Almost Never<br>◯ Sometimes<br>◯ Often<br>◯ Almost Always

**Getting Along with Others (in the past one month)**	
My child has trouble getting along with other kids:	◯ Never<br>◯ Almost Never<br>◯ Sometimes<br>◯ Often<br>◯ Almost Always
Other kids do not want to be friends with my child:	◯ Never<br>◯ Almost Never<br>◯ Sometimes<br>◯ Often<br>◯ Almost Always
Other kids tease my child:	◯ Never<br>◯ Almost Never<br>◯ Sometimes<br>◯ Often<br>◯ Almost Always
My child cannot do things that other kids my age can do:	◯ Never<br>◯ Almost Never<br>◯ Sometimes<br>◯ Often<br>◯ Almost Always

It is hard for my child to keep up when he/she plays with other kids:	◯ Never<br>◯ Almost Never<br>◯ Sometimes<br>◯ Often<br>◯ Almost Always
**School (in the past one month)**	
It is hard for my child to pay attention in class:	◯ Never<br>◯ Almost Never<br>◯ Sometimes<br>◯ Often<br>◯ Almost Always
My child forgets things:	◯ Never<br>◯ Almost Never<br>◯ Sometimes<br>◯ Often<br>◯ Almost Always
My child has trouble keeping up with his/her schoolwork:	◯ Never<br>◯ Almost Never<br>◯ Sometimes<br>◯ Often<br>◯ Almost Always
My child misses school because of not feeling well:	◯ Never<br>◯ Almost Never<br>◯ Sometimes<br>◯ Often<br>◯ Almost Always
My child misses school to go to the doctor or hospital:	◯ 1. Never<br>◯ 2. Almost Never<br>◯ 3. Sometimes<br>◯ 4. Often<br>◯ 5. Almost Always

Appendix 3: 36-Item Short Form Survey (SF-36)

SF-36 QUESTIONNAIRE

Name:____________________ **Ref. Dr:**__________________ **Date:** _______

ID#: _______________ **Age:** _______ **Gender: M / F**

Please answer the 36 questions of the **Health Survey** completely, honestly, and without interruptions.

GENERAL HEALTH:

In general, would you say your health is:

- Excellent
- Very Good
- Good
- Fair
- Poor

Compared to one year ago, how would you rate your health in general now?

- Much better now than one year ago
- Somewhat better now than one year ago
- About the same
- Somewhat worse now than one year ago
- Much worse than one year ago

LIMITATIONS OF ACTIVITIES:

The following items are about activities you might do during a typical day. Does your health now limit you in these activities? If so, how much?

Vigorous activities, such as running, lifting heavy objects, participating in strenuous sports.

- Yes, Limited a lot
- Yes, Limited a Little
- No, Not Limited at all

Moderate activities, such as moving a table, pushing a vacuum cleaner, bowling, or playing golf

- Yes, Limited a Lot
- Yes, Limited a Little
- No, Not Limited at all

Lifting or carrying groceries

- Yes, Limited a Lot
- Yes, Limited a Little
- No, Not Limited at all

Climbing several flights of stairs

- Yes, Limited a Lot
- Yes, Limited a Little
- No, Not Limited at all

Climbing one flight of stairs

- Yes, Limited a Lot
- Yes, Limited a Little
- No, Not Limited at all

Bending, kneeling, or stooping

- Yes, Limited a Lot
- Yes, Limited a Little
- No, Not Limited at all

Walking more than a mile

- Yes, Limited a Lot
- Yes, Limited a Little
- No, Not Limited at all

Walking several blocks

- Yes, Limited a Lot
- Yes, Limited a Little
- No, Not Limited at all

Walking one block

- Yes, Limited a Lot
- Yes, Limited a Little
- No, Not Limited at all

Bathing or dressing yourself
Yes, Limited a Lot Yes, Limited a Little No, Not Limited at all

PHYSICAL HEALTH PROBLEMS:
During the past 4 weeks, have you had any of the following problems with your work or other regular daily activities as a result of your physical health?

Cut down the amount of time you spent on work or other activities
Yes No

Accomplished less than you would like
Yes No

Were limited in the kind of work or other activities
Yes No

Had difficulty performing the work or other activities (for example, it took extra effort)
Yes No

EMOTIONAL HEALTH PROBLEMS:
During the past 4 weeks, have you had any of the following problems with your work or other regular daily activities as a result of any emotional problems (such as feeling depressed or anxious)?

Cut down the amount of time you spent on work or other activities
Yes No

Accomplished less than you would like
Yes No

Didn't do work or other activities as carefully as usual
Yes No

SOCIAL ACTIVITIES:
Emotional problems interfered with your normal social activities with family, friends, neighbors, or groups?

Not at all Slightly Moderately Severe Very Severe

PAIN:
How much bodily pain have you had during the past 4 weeks?

None Very Mild Mild Moderate Severe Very Severe

During the past 4 weeks, how much did pain interfere with your normal work (including both work outside the home and housework)?

Not at all A little bit Moderately Quite a bit Extremely

ENERGY AND EMOTIONS:

These questions are about how you feel and how things have been with you during the last 4 weeks. For each question, please give the answer that comes closest to the way you have been feeling.

Did you feel full of pep?

- All of the time
- Most of the time
- A good Bit of the Time
- Some of the time
- A little bit of the time
- None of the Time

Have you been a very nervous person?

- All of the time
- Most of the time
- A good Bit of the Time
- Some of the time
- A little bit of the time
- None of the Time

Have you felt so down in the dumps that nothing could cheer you up?

- All of the time
- Most of the time
- A good Bit of the Time
- Some of the time
- A little bit of the time
- None of the Time

Have you felt calm and peaceful?

- All of the time
- Most of the time
- A good Bit of the Time
- Some of the time
- A little bit of the time
- None of the Time

Did you have a lot of energy?

- All of the time
- Most of the time
- A good Bit of the Time
- Some of the time
- A little bit of the time
- None of the Time

Have you felt downhearted and blue?

- All of the time
- Most of the time
- A good Bit of the Time
- Some of the time
- A little bit of the time
- None of the Time

Did you feel worn out?

- All of the time
- Most of the time
- A good Bit of the Time
- Some of the time
- A little bit of the time
- None of the Time

Have you been a happy person?

- All of the time
- Most of the time
- A good Bit of the Time
- Some of the time
- A little bit of the time
- None of the Time

Did you feel tired?

- All of the time
- Most of the time
- A good Bit of the Time
- Some of the time
- A little bit of the time
- None of the Time

SOCIAL ACTIVITIES:
During the past 4 weeks, how much of the time has your physical health or emotional problems interfered with your social activities (like visiting with friends, relatives, etc.)?

- All of the time
- Most of the time
- Some of the time
- A little bit of the time
- None of the Time

GENERAL HEALTH:
How true or false is each of the following statements for you?

I seem to get sick a little easier than other people
◯ Definitely true ◯ Mostly true ◯ Don't know ◯ Mostly false ◯ Definitely false

I am as healthy as anybody I know
◯ Definitely true ◯ Mostly true ◯ Don't know ◯ Mostly false ◯ Definitely false

I expect my health to get worse
◯ Definitely true ◯ Mostly true ◯ Don't know ◯ Mostly false ◯ Definitely false

My health is excellent
◯ Definitely true ◯ Mostly true ◯ Don't know ◯ Mostly false ◯ Definitely false

Appendix 4: Qualitative Analysis Interview Guide

Group 1 Interview Guide for Parents (participant ages 5-12 and 13-17)

Introductory remarks: To be given by the moderator

*Slight alternatives will be given for the different subgroups of this study: children, parents (ages 5-8 and 8-17), young adults (ages 18-25), female adults of reproductive age (ages 15-25).

Thank you for joining us today for this interview. We are here today to learn from you how children living with corrected congenital heart disease feel about their quality of life. We are interested in specific experiences that might not be addressed by our survey, and in the perspectives of parents on life with a child with congenital heart disease after surgery. We plan to use these interviews to supplement our more general survey, and your responses will inform the results of our survey and explain some of the trends we find. We also plan to use your responses to better identify areas of need for intervention, including medical, economic and social, to improve long-term quality of life post-surgery for patients living with corrected CHD.

<u>You</u> have been invited to participate in our study for an interview, among other participants and families with experiences that we would like to better understand. The format is an individual interview where all responses will be kept anonymous. We hope for you to share your opinions and experiences on the topics we discuss today, ultimately allowing us to build a better congenital heart disease health program.

<u>There are several important rules for this interview:</u>

1. There are no right or wrong answers. We simply wish to hear your opinions on the matters we are to discuss.
2. All of the opinions and statements from today's discussion will remain confidential and will not be repeated outside of this interview.
3. For your protection and to preserve anonymity, please choose a fake name which cannot be traced back to you. Every time you speak into the digital audio recorder, please state that "it is [your chosen fake name]" so that we know who is speaking when we review the record later.
4. The study team is the only group of people who will have access to the tape. Nobody outside this room will be able to trace your comments back to you.
5. Please speak one-by-one (for dyadic interviews with both parents and children). Do not speak when someone else is talking. Also, speak clearly and loudly so the recorder can pick up what is said.

As the moderator, I am here to ask questions and listen. My role is to guide the discussion to collect the information most useful for the researchers.

Questions: To be given by the moderator

1. What were your expectations of your child's heart surgery and how were they met/unmet? (*Individual*)
2. Tell me what you know about your child's heart illness (congenital heart disease)?
3. Tell me about the symptoms your children experience related to their heart condition or surgical procedure? (*Individual*)
4. In what areas of your life do you feel like your child's heart condition and/or their surgery affect you the most (family, school, village, work, physical health, mental health, other)? (*All SEM levels*)

5. How do you feel like your child is treated by your family and friends? Do they understand the conditions and the needs of your child? (*Interpersonal*)
6. Tell me what most concerns you due to your child's health? (*All SEM levels*)
7. Do you feel that your child's health-related needs are met by the resources available? (*organizational, community, public policy)*
8. Are you worried that your child will need additional surgery, what are the issues around this possible future surgery that you are the most worried about?

Concluding Question:
Is there anything about this that you want me to know that I didn't ask?

Group 2 Interview Guide for Adolescents and Emancipated minors (participant ages 13-17)

Introductory remarks: To be given by the moderator

Thank you for joining us today for this interview. We are here today to learn from you how adolescents living with corrected congenital heart disease feel about their quality of life. We are interested in specific experiences that might not be addressed by our survey, and in the perspectives of both adolescents and their parents on life with a congenital heart disease after surgery. We plan to use these interviews to supplement our more general survey, and your responses will inform the results of our survey and explain some of the trends we find. We also plan to use your responses to better identify areas of need for intervention, including medical, economic and social, to improve long-term quality of life post-surgery for patients living with corrected CHD.

You have been invited to participate in our study for an interview, among other participants and families with experiences that we would like to better understand. The format is an individual interview where all responses will be kept anonymous. We hope for you to share your opinions and experiences on the topics we discuss today, ultimately allowing us to build a better congenital heart disease health program.

There are several important rules for this interview:

1. There are no right or wrong answers. We simply wish to hear your opinions on the matters we are to discuss.
2. All of the opinions and statements from today's discussion will remain confidential and will not be repeated outside of this interview.
3. For your protection and to preserve anonymity, please choose a fake name which cannot be traced back to you. Every time you speak into the digital audio recorder, please state that "it is [your chosen fake name]" so that we know who is speaking when we review the record later.
4. The study team is the only group of people who will have access to the tape. Nobody outside this room will be able to trace your comments back to you.
5. Please speak one-by-one (for dyadic interviews with both parents and children). Do not speak when someone else is talking. Also, speak clearly and loudly so the recorder can pick up what is said.

As the moderator, I am here to ask questions and listen. My role is to guide the discussion to collect the information most useful for the researchers.

Questions: To be given by the moderator

1. What were your expectations of your heart surgery and how were they met/unmet? (*Individual*)
2. Tell me what you know about your heart disease(congenital heart disease)?
3. Tell me about the symptoms you experience related to your heart condition or surgical procedure? (*Individual*)
4. In what areas of your life do you feel like your heart condition and/or surgery affect you the most (family, school, village, gender, work, physical health, mental health, other)? (*All SEM levels*)
5. How do you feel like you are treated by your family and friends? Do they understand the conditions and your needs? (*Interpersonal*)
6. Tell me what most concerns you due to your health? (*All SEM levels*)
7. Do you feel that your or your child's health-related needs are met by the resources available? (*organizational, community, public policy)*
   Are you worried that you will need additional surgery, what are the issues around this possible future surgery that you are the most worried about?

Group 3 Interview Guide for Young Adults (participant ages 18-25)

Introductory remarks: To be given by the moderator

Thank you for joining us today for this interview. We are here today to learn from young adults living with corrected congenital heart disease feel about their quality of life. We are interested in specific experiences that might not be addressed by our survey and how congenital heart disease fully impacts the lives of young adults as they become more independent and seek jobs and families. We plan to use these interviews to supplement our more general survey, and your responses will inform the results of our survey and explain some of the trends we find. We also plan to use your responses to better identify areas of need for intervention, including medical, economic and social, to improve long-term quality of life post-surgery for patients living with corrected CHD.

<u>You</u> have been invited to participate in our study for an interview, among other participants with experiences that we would like to better understand. The format is an individual interview where all responses will be kept anonymous. We hope for you to share your opinions and experiences on the topics we discuss today, ultimately allowing us to build a better congenital heart disease health program.

<u>There are several important rules for this interview:</u>

1. There are no right or wrong answers. We simply wish to hear your opinions on the matters we are to discuss.
2. All of the opinions and statements from today's discussion will remain confidential and will not be repeated outside of this interview.
3. For your protection and to preserve anonymity, please choose a fake name which cannot be traced back to you. Every time you speak into the digital audio recorder, please state that "it is [your chosen fake name]" so that we know who is speaking when we review the record later.
4. The study team is the only group of people who will have access to the tape. Nobody outside this room will be able to trace your comments back to you.
5. Please speak one-by-one (for dyadic interviews with both parents and children). Do not speak when someone else is talking. Also, speak clearly and loudly so the recorder can pick up what is said.

As the moderator, I am here to ask questions and listen. My role is to guide the discussion to collect the information most useful for the researchers.

<u>Questions:</u>

1. What were your expectations of your heart surgery and how were they met/unmet? (*Individual*)
2. Tell me what you know about your heart illness (congenital heart disease)?
3. Tell me about the symptoms experience related to their heart condition or surgical procedure? (*Individual*)
4. In what areas of your life do you feel like heart condition and/or your surgery affect you the most (family, school, village, work, physical health, mental health, other)? (*All SEM levels*)
5. How do you feel like you are treated by your family and friends? Do they understand the conditions and your needs? (*Interpersonal*)
6. Tell me what most concerns you due to your health? (*All SEM levels*)
7. Do you feel that your health-related needs are met by the resources available? (*organizational, community, public policy)*
8. Are you worried that you will need additional surgery, what are the issues around this possible future surgery that you are the most worried about?
9. Does your heart condition affect your ability to find employment? (*All SEM levels, particularly organizational and community*)
10. How do you think your heart condition affects your plan to marry and have a family? Why do you feel this way? (*Individual*, *interpersonal, community)*

Additional Questions for Female Participants:

1. What has your doctor told you about your heart disease and pregnancy? Has a doctor ever told you that your heart is not strong enough to support a pregnancy?
    a. How has this information impacted your desire to have children, if at all? (*Individual)*
    b. What is your experience with being offered birth control methods such as condoms, medications, or an IUD (intrauterine device)? (*organizational, community*)
    c. How has the information about the strength of your heart in regards to supporting a pregnancy impacted your decision to be on birth control? (*Individual)*
    d. Do you think there is prejudice or judgment directed against women of childbearing age who have heart disease and may not have hearts strong enough to survive a pregnancy?
        - If so, from who? – Parents? Husbands? In-laws? Friends/Neighbors? Doctors/ Healthcare providers? (*Interpersonal, organizational, community, public policy)*
    e. Are there barriers for women with heart disease to obtaining birth control therapies? (*Organizational, community, public policy)*
        - If so, what are the barriers?
            - Cost of medicine/ treatments?
            - Cost of travel?
            - Distance from health centers/ pharmacies?
            - Fear of medications?
            - Judgment by family/ friends?
